# Myo-Inositol Plus Selenium vs. Selenium Alone in Hashimoto’s Thyroiditis with Subclinical Hypothyroidism: A Systematic Review and Updated Meta-Analysis with Trial Sequential Analysis

**DOI:** 10.3390/jcm15093179

**Published:** 2026-04-22

**Authors:** Pavel Stanchev, Maria Kraeva, Petar-Preslav Petrov, Plamen Penchev

**Affiliations:** 1 Clinic of Endocrinology and Metabolic Diseases, St. George University Hospital, Medical University of Plovdiv, 4000 Plovdiv, Bulgaria; dr.p.stanchev@abv.bg; 2 Department of Otorhinolaryngology, Medical University of Plovdiv, 4000 Plovdiv, Bulgaria; kraevamaria93@gmail.com; 3 Department of Anatomy, Histology and Embryology, Medical University of Plovdiv, 4000 Plovdiv, Bulgaria; dr.petar.preslav@gmail.com; 4 Faculty of Medicine, Medical University of Plovdiv, 4000 Plovdiv, Bulgaria

**Keywords:** Hashimoto’s thyroiditis, subclinical hypothyroidism, myo-inositol, selenium, meta-analysis, thyroid autoimmunity

## Abstract

**Introduction**: Hashimoto’s thyroiditis (HT) is the leading cause of hypothyroidism in iodine-sufficient regions and often presents with subclinical hypothyroidism. Selenium (Sel) has immunomodulatory effects, while myo-inositol (MI) may enhance thyroid-stimulating hormone (TSH) signaling. This study evaluated whether adding myo-inositol to selenium provides additional benefit compared with selenium alone in these patients. **Methods**: A systematic search of PubMed, Web of Science, and the Cochrane Library was conducted from inception to 7 March 2026. Studies comparing myo-inositol plus selenium (MI + Sel) with Sel monotherapy were included. Standardized mean differences (SMDs) with 95% confidence intervals (CIs) were pooled using a frequentist random-effects model. Outcomes of interest included TSH, free T3 and T4, thyroglobulin antibodies (TgAb), and thyroid peroxidase antibodies (TPOAb). Trial sequential analysis (TSA) was performed to assess the robustness of significant findings. **Results**: Three studies involving 288 patients were included (151 receiving MI + Sel and 137 receiving Sel alone). Combination therapy significantly reduced TSH levels compared with Sel monotherapy (SMD −1.26; 95% CI −1.51 to −1.00; *p* < 0.01; I^2^ = 0%), and TSA suggested that this finding may be robust, although the evidence is limited by the small number of studies. TgAb levels were also significantly reduced (SMD −0.51; 95% CI −0.78 to −0.24; *p* < 0.01; I^2^ = 0%); however, TSA indicated a potential risk of type I error. No significant differences were observed for T3 (SMD 0.15; 95% CI −0.09 to 0.38; *p* = 0.22; I^2^ = 7%), T4 (SMD −0.01; 95% CI −0.72 to 0.69; *p* = 0.97; I^2^ = 88%), or TPOAb (SMD −0.18; 95% CI −0.44 to 0.09; *p* = 0.20; I^2^ = 0%). **Conclusions**: MI combined with Sel was associated with a significant reduction in TSH levels compared with Sel alone in patients with HT and subclinical hypothyroidism, suggesting a potential therapeutic benefit. However, given the limited number of studies, these findings should be interpreted with caution. Further large randomized controlled trials are required to confirm the effects on thyroid function and autoimmunity.

## 1. Introduction

Hashimoto’s thyroiditis (HT) is the most common autoimmune thyroid disorder and the leading cause of hypothyroidism in iodine-sufficient regions worldwide [1]. It is characterized by chronic autoimmune-mediated destruction of the thyroid gland and the presence of circulating thyroid autoantibodies, particularly thyroid peroxidase antibodies (TPOAb) and thyroglobulin antibodies (TgAb) [2]. The disease frequently presents with subclinical hypothyroidism, defined by elevated thyroid-stimulating hormone (TSH) levels with normal free thyroxine (fT4), and may progress to overt hypothyroidism over time [3]. Subclinical hypothyroidism has been associated with metabolic disturbances, increased cardiovascular risk, and reduced quality of life, highlighting the need for effective management strategies [2,3].

Selenium (Sel), an essential trace element, plays a key role in thyroid hormone metabolism and antioxidant defense. Due to its immunomodulatory properties, selenium supplementation has been investigated as an adjunctive therapy in autoimmune thyroid disease, with some studies reporting reductions in thyroid autoantibody levels and modest improvements in thyroid function, although results remain inconsistent [4,5].

Myo-inositol (MI), a naturally occurring compound involved in intracellular signaling pathways, has recently emerged as a potential modulator of thyroid function. It is thought to enhance TSH signaling and support thyroid hormone synthesis, with emerging clinical evidence suggesting a beneficial effect on thyroid function parameters [6].

Clinical studies have evaluated the combined use of myo-inositol and selenium in patients with autoimmune thyroiditis and subclinical hypothyroidism, reporting improvements in thyroid function and antibody levels, although findings remain heterogeneous [5,6,7].

Despite these promising findings, the available evidence remains limited, and there is currently no consensus regarding the clinical benefit of combination therapy over selenium alone. Given the high prevalence of subclinical hypothyroidism and the lack of standardized adjunctive treatment strategies, a comprehensive synthesis of the existing evidence is warranted. Therefore, the present systematic review and meta-analysis aimed to evaluate the efficacy of myo-inositol combined with selenium compared with selenium monotherapy in patients with Hashimoto’s thyroiditis and subclinical hypothyroidism.

## 2. Methods

### 2.1. Eligibility Criteria

This systematic review and meta-analysis followed the Cochrane Handbook for Systematic Reviews of Interventions and the Preferred Reporting Items for Systematic Reviews and Meta-Analysis Statement [8,9] (PRISMA checklist Supplement). This meta-analysis did not require Institutional Review Board approval because it used data from previously published and publicly available articles. Studies that met all the following criteria were included in the meta-analysis: (1) observational studies (case–control, cohort, cross-sectional) or randomized trials; (2) studies with adult patients with a confirmed diagnosis of Hashimoto’s thyroiditis (based on ultrasonographic patterns and/or positive TPOAb/TgAb) and concomitant subclinical hypothyroidism; (3) combined supplementation of MI plus Sel, regardless of dosage, frequency, or duration of treatment; (4) Studies with control group receiving SE monotherapy; (5) Studies reporting at least one of the following outcomes: primary: serum thyroid-stimulating hormone (TSH) levels, secondary: free thyroxine (fT4), free triiodothyronine (fT3), thyroid peroxidase antibodies (TPOAb), or thyroglobulin antibodies (TgAb). Studies were excluded if they met one of the following criteria: (1) Patients with overt hypothyroidism, hyperthyroidism, or non-autoimmune thyroid disorders, unless data for the subclinical HT subgroup were reported separately; (2) Pediatric or adolescent populations (<18 years); (3) Studies evaluating MI or Se as monotherapies without a combination arm, or studies lacking a proper comparative control group; (4) overlapping populations; (5) Case reports or series, editorials, letters, conference abstracts without full-text availability, and animal or in vitro models; (6) No outcome of interest. This systematic review and meta-analysis was registered with the International Prospective Register of Systematic Reviews (PROSPERO) under the ID “CRD420261345957”.

### 2.2. Search Strategy and Data Extraction

We systematically searched PubMed, Web of Science, and Cochrane Central from inception to 7 March 2026 with the following search strategy: (“Hashimoto Disease” [Mesh] OR “Hashimoto thyroiditis” OR “autoimmune thyroiditis”) AND (hypothyroidism OR “subclinical hypothyroidism”) AND (“myo-inositol” OR inositol OR selenium OR “selenium supplementation” OR selenomethionine) AND (“selenium alone” OR selenium OR “selenium monotherapy”). Restrictions were applied to only English-language articles. Gray literature was excluded. We manually searched the references of all included studies to identify any additional studies. Two authors (P.S. and P.P.) independently extracted the data using predefined search criteria, quality assessment methods, and Rayyan 4.2.1 software [10]. Any disagreements between these authors were resolved through consensus. For continuous outcomes, final values were preferentially extracted. When both final and change-from-baseline values were reported, final values were used for consistency. If data were presented at multiple time points, the longest follow-up was selected. When required, standard deviations were calculated from available data or estimated using established methods. Authors were contacted when necessary; if data remained unavailable, studies were included qualitatively but excluded from quantitative synthesis.

### 2.3. Endpoints and Subgroup Analyses

The meta-analysis included TSH levels, fT4, fT3, TPOAb, or TgAb endpoints. Additionally, we conducted a subgroup analysis for TSH, fT4, and fT3 based on study design.

### 2.4. Quality Assessment

For non-randomized studies, the risk of bias was assessed using the Cochrane Collaboration’s tool for assessing the risk of bias in non-randomized studies of interventions (ROBINS-I) [11]. The ROBINS-I tool categorizes the risk of bias as low, moderate, serious, or critical. The risk of bias for the RCTs was assessed using the Cochrane Collaboration’s tool for assessing the risk of bias in randomized studies of interventions (RoB 2) [12]. The RoB 2 tool categorizes the risk of bias as low, some concerns, or high. Two authors (P.S. and P.P.) independently performed the assessments, resolving disagreements through consensus. Publication bias was evaluated using contour-enhanced funnel plots with the trim-and-fill method, which allows for a better interpretation of asymmetry related to statistical significance thresholds. Additional methods, such as *p*-curve or *p*-uniform analysis, were not feasible due to the absence of reported exact *p*-values or test statistics in all included studies. Following the Cochrane guidelines, the Egger test was not performed because fewer than 10 studies were included in the meta-analysis [9].

### 2.5. Statistical Analysis

Standardized mean difference (SMD) with 95% confidence intervals (CI) was computed to compare effects for continuous endpoints using the restricted maximum-likelihood estimator random-effects method [13,14]. A random-effects model was applied for all outcomes to account for demographic and methodological variability. Heterogeneity was assessed using the I^2^ statistic and the Cochran Q test. The χ^2^ test was used to assess subgroup differences. Two-sided *p*-values < 0.05 were regarded as statistically significant. Subgroup analyses were performed based on the study design to minimize the risk of selection bias. Leave-one-out (LOO) sensitivity analyses were also conducted to assess the consistency of the findings. A Baujat plot was generated to identify studies contributing most to heterogeneity and their influence on the overall meta-analysis results. This diagnostic tool visually represents the balance between a study’s contribution to heterogeneity (x-axis) and its weight in the meta-analysis (y-axis), aiding in the interpretation of outlier or highly influential studies. Statistical analysis was performed using R software version 4.3.1 with the packages “metafor” and “meta” [15]. 

### 2.6. Trial Sequential Analysis (TSA)

Trial Sequential Analysis (TSA) was performed to assess the robustness of the meta-analysis results and to control for risks of type I and type II errors due to sparse data and repeated testing [16]. TSA was conducted using a two-sided α of 5% and power of 80%. For continuous outcomes, the required information size (RIS) was calculated based on the observed standard deviation and a clinically meaningful effect size derived from the pooled estimates, with adjustment for heterogeneity using a random-effects model. Cumulative Z-curves were plotted to determine whether the evidence crossed monitoring boundaries for statistical significance.

## 3. Results

### 3.1. Study Selection and Baseline Characteristics

**Figure 1:**
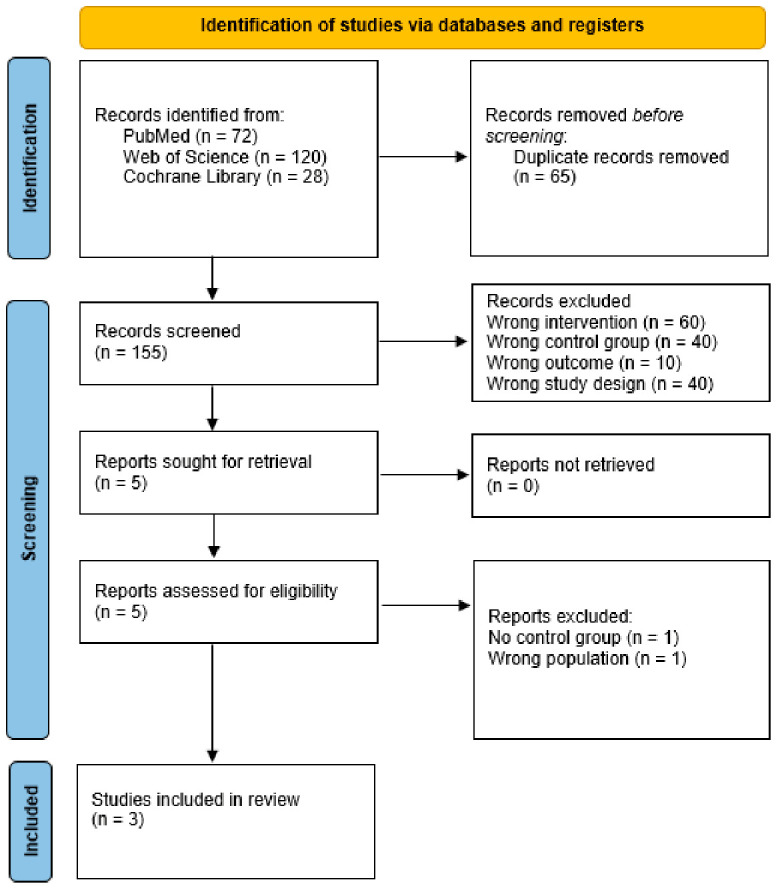
PRISMA flow diagram and study selection.

The search strategy yielded a total of 220 results. After removing duplicate records and unrelated articles or abstracts, the remaining 5 studies were fully reviewed to determine whether they met the inclusion and exclusion criteria (Figure 1). Two studies were excluded after full-text review: one due to a lack of a control group and one due to the inclusion of patients without subclinical hypothyroidism. Three studies were included, with 288 patients [7,17,18]. Of those, 151 patients (52%) were treated with MI + Sel and were included in our analyses. The mean age of the population was 41 years. Study characteristics are presented in Table 1.

### 3.2. Pooled Analyses of the Included Studies

#### 3.2.1. TSH

**Figure 2:**
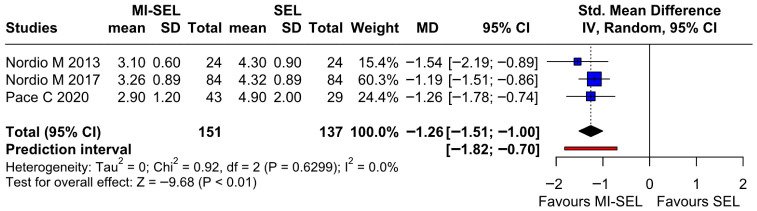
Forest plot comparing the effect of MI-SEL versus SEL monotherapy on TSH levels. The pooled SMD indicates a significant reduction in TSH levels favoring the combined MI-SEL treatment [7,17,18].

**Figure 3:**
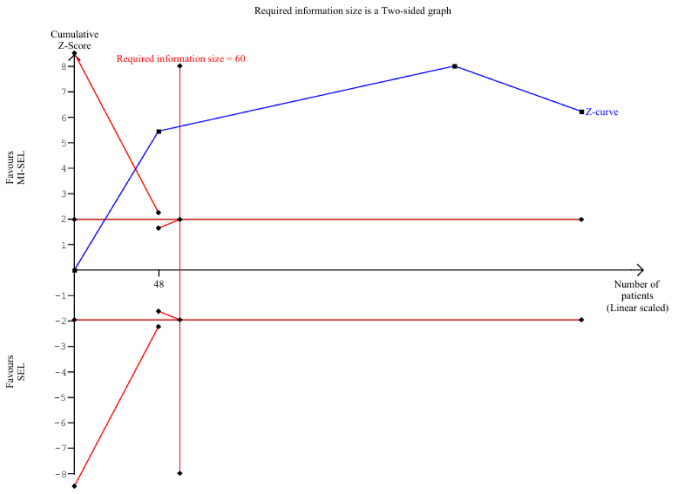
TSA for TSH reduction comparing MI-SEL versus SEL. The cumulative Z-curve (blue line) crosses the trial sequential monitoring boundary and exceeds the required information size (RIS = 60), indicating a statistically significant benefit for the combined treatment.

The meta-analysis revealed that the combined treatment (MI-Sel) was significantly more effective at reducing TSH levels compared to Sel monotherapy (SMD—1.26; 95% CI [−1.51; −1]; *p* < 0.01; I^2^ = 0%) (Figure 2). TSA showed that the cumulative Z-curve crossed the monitoring boundary for benefit, suggesting that the observed effect is unlikely to be due to random error; however, this finding should be interpreted in the context of the limited number of included studies (Figure 3). LOO sensitivity analysis demonstrated that the overall effect size remained consistent across all iterations, with statistical significance preserved throughout (Supplementary Figure S1). This suggests that no single study had a dominant influence on the pooled estimate, although the small number of included studies limits the reliability of this assessment. The Baujat plot identified the studies by Nordio M. & Basciani, 2017 [7] and Nordio M. & Pajalich, 2013 [17] as potentially influential, contributing substantially to the overall result and heterogeneity (Supplementary Figure S2).

#### 3.2.2. fT4

**Figure 4:**
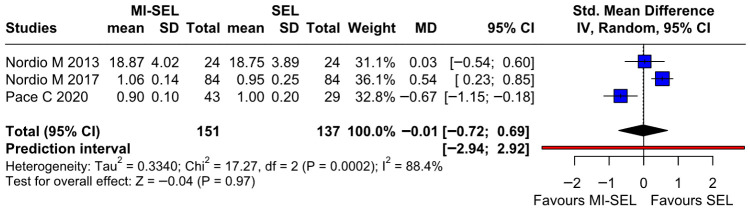
Forest plot comparing the effect of MI-SEL versus SEL monotherapy on fT4 levels. The pooled results show no significant difference between the two treatments [7,17,18].

The meta-analysis revealed no significant differences between the combined treatment (MI-Sel) and Sel monotherapy in reducing fT4 (SMD—0.01; 95% CI [−0.72; 0.69]; *p* = 0.97; I^2^ = 88%) (Figure 4). LOO sensitivity analysis demonstrated that the overall effect size remained consistent across all iterations. The result remained non-significant across all iterations (Supplementary Figure S3). This suggests that no single study has a disproportionate influence on the overall outcome. The Baujat plot identified the studies by Nordio M. & Basciani, 2017 [7] and Pace et al. 2020 [18] as potentially influential, contributing substantially to the overall result and heterogeneity (Supplementary Figure S4).

#### 3.2.3. fT3

**Figure 5:**
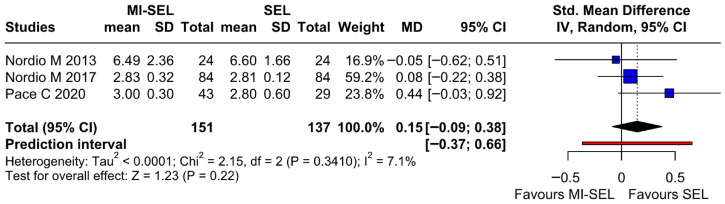
Forest plot comparing the effect of MI-SEL versus SEL monotherapy on fT3 levels. The pooled results show no significant difference between the two treatments [7,17,18].

The meta-analysis revealed no significant differences between the combined treatment (MI-Sel) and Sel monotherapy in reducing fT3 (SMD 0.15; 95% CI [−0.09; 0.38]; *p* = 0.22; I^2^ = 7%) (Figure 5). The LOO analysis showed that the results remained stable across all iterations (Supplementary Figure S5). This suggests that no single study has a disproportionate influence on the overall outcome. The Baujat plot identified the study by Pace et al. 2020 [18] as potentially influential, contributing substantially to the overall heterogeneity (Supplementary Figure S6).

#### 3.2.4. TgAb

**Figure 6:**
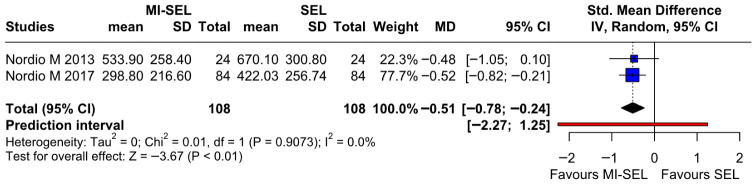
Forest plot comparing the effect of MI-SEL versus SEL monotherapy on TgAb levels. The pooled SMD indicates a significant reduction in TgAb levels, favoring the combined MI-SEL treatment [7,17].

**Figure 7:**
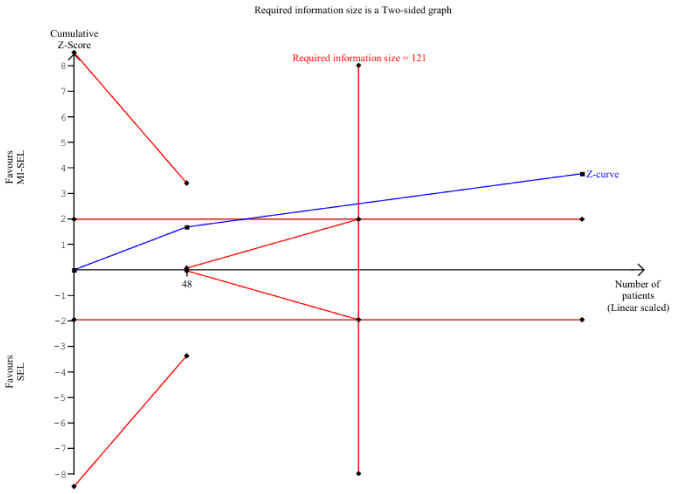
TSA on TgAb levels. TSA showed that although the cumulative Z-curve crossed the conventional significance boundary, it did not cross the trial sequential monitoring boundary for benefit, indicating that the evidence remains inconclusive and may be at risk of type I error.

The combined treatment (MI-Sel) was significantly more effective in reducing TgAb levels compared to Sel monotherapy (SMD—0.51; 95% CI [−0.78; −0.24]; *p* < 0.01; I^2^ = 0%) (Figure 6). TSA indicated a statistically significant difference between the groups; however, as the cumulative Z-curve did not cross the trial sequential monitoring boundary for benefit, the observed significance may be at risk of type I error (Figure 7). The overall effect size remained consistent across all iterations, and the result remained significant in all cases as demonstrated by the LOO analysis (Supplementary Figure S7). This suggests that no single study has a disproportionate influence on the overall outcome. The Baujat plot identified the studies by Nordio M. & Basciani, 2017 [7] and Nordio M. & Pajalich, 2013 [17] as potentially influential, contributing substantially to the overall result and heterogeneity (Supplementary Figure S8).

#### 3.2.5. TPOAb

**Figure 8:**
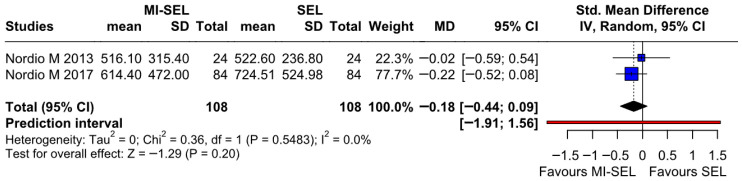
Forest plot comparing the effect of MI-SEL versus SEL monotherapy on TPOAb levels. The pooled results show no significant difference between the two treatments [7,17].

The meta-analysis revealed no significant differences between the combined treatment (MI-Sel) and Sel monotherapy in reducing TPOAb (SMD −0.18; 95% CI [−0.44; 0.09]; *p* = 0.20; I^2^ = 0%) (Figure 8). LOO analysis indicated that the overall effect size remained consistent across all iterations and the result remained non-significant in all cases (SMD −0.18; 95% CI [−0.44; 0.09]; *p* = 0.20; I^2^ = 0%) (Supplementary Figure S9). The Baujat plot identified the studies by Nordio M. & Basciani, 2017 [7] and Nordio M. & Pajalich, 2013 [17] as potentially influential, contributing substantially to the overall result and heterogeneity (Supplementary Figure S10).

### 3.3. Subgroup Analyses

#### 3.3.1. TSH Based on Study Design

To evaluate the impact of study methodology on the outcomes, a subgroup analysis was performed comparing randomized controlled trials (RCTs) and observational studies. The pooled analysis of the two RCTs demonstrated a significant reduction in TSH levels in favor of MI–Sel (SMD: −1.26; 95% CI [−1.55; −0.96]; *p* < 0.01). The observational study reported a similar direction and magnitude of effect (SMD: −1.26; 95% CI [−1.78; −0.74]; *p* < 0.01). The test for subgroup differences showed no statistically significant difference between study designs (χ^2^ = 0.00, df = 1, *p* = 0.99). However, this finding should be interpreted with caution given the small number of studies and the inclusion of one observational study with a serious risk of bias (Supplementary Figure S11).

#### 3.3.2. fT4 Based on Study Design

A subgroup analysis was performed to explore the influence of study design on the results. The observational study reported a significant reduction in fT4 levels in the MI–Sel group, whereas the pooled RCTs showed no significant difference between groups. The test for subgroup differences was statistically significant (χ^2^ = 8.29, df = 1, *p* = 0.004). However, given the limited number of studies and the presence of only a single observational study, this finding should be considered exploratory and interpreted with caution (Supplementary Figure S12).

#### 3.3.3. fT3 Based on Study Design

A subgroup analysis was conducted to assess if the study methodology influenced the outcomes for fT3 (Supplementary Figure S13). The study by Pace et al. 2020 [18] reported a non-significant trend toward higher fT3 levels in the SEL group (SMD 0.44; 95% CI [−0.03; 0.92]; *p* = 0.07). The pooled analysis of the two RCTs (Nordio M. & Pajalich, 2013 [17], Nordio M. & Basciani, 2017 [7]) showed a near-zero effect (SMD 0.05; 95% CI [−0.21; 0.32]; *p* = 0.68) with no internal heterogeneity (I^2^ = 0%). The test for subgroup differences revealed no significant difference between the observational and RCT groups (χ2 = 1.98, df = 1, *p* = 0.16). This suggests that the lack of effect on fT3 levels appears consistent across different study designs.

### 3.4. Quality Assessment

The one included observational study was assessed as having a serious risk of bias based on the ROBINS-I tool. The detailed evaluation is presented in Supplementary Figure S14. Among the two included RCT studies, one was assessed as having some concerns surrounding risk of bias, and one as having a low risk of bias based on the RoB 2 tool. The detailed evaluation is presented in Supplementary Figure S15. Publication bias was evaluated using contour-enhanced trim-and-fill funnel-plot analyses, plotting individual study weights against point estimates. The funnel plots for the outcomes did show some asymmetry, but given the small number of included studies, visual interpretation is limited (Supplementary Figures S16–S20).

## 4. Discussion

This systematic review and meta-analysis of three studies and 288 patients evaluated the efficacy of MI–Sel compared with Sel monotherapy in patients with Hashimoto’s thyroiditis and subclinical hypothyroidism. Two additional studies identified in the literature search evaluated MI + Sel without a comparator group and reported improvements in thyroid function parameters and antibody levels. Although these findings are consistent with our results, the lack of control groups limits causal inference and precluded their inclusion in the meta-analysis. The pooled analysis demonstrated that the combined therapy significantly reduced TSH levels compared with selenium alone [7,17,18]. In contrast, no significant differences were observed for fT3, fT4, or TPOAb levels, while TgAb levels were significantly reduced in the combined treatment group, although trial sequential analysis suggested that this latter finding should be interpreted cautiously. Overall, these results suggest that the addition of myo-inositol to selenium may provide a meaningful improvement in thyroid function, primarily reflected by reductions in TSH levels.

The most notable finding of the present analysis is the significant reduction in TSH levels associated with MI–Sel therapy. TSH is the most sensitive marker of thyroid function and is typically elevated in subclinical hypothyroidism as a compensatory response to impaired thyroid hormone production. The observed reduction in TSH suggests an improvement in thyroid responsiveness or hormone synthesis with combination therapy. Importantly, trial sequential analysis showed that the cumulative evidence crossed both the conventional significance boundary and the monitoring boundary for benefit, suggesting that the observed effect is unlikely to be due to random error. However, this finding should be interpreted in the context of the limited number of included studies. Furthermore, leave-one-out sensitivity analysis showed consistent effect estimates across iterations, while subgroup analysis demonstrated a similar direction of effect across randomized controlled trials [7,17] and the observational study [18]. Taken together, these findings suggest a potentially consistent association between MI–Sel supplementation and TSH reduction, although the strength of the evidence remains limited.

The biological rationale for combining myo-inositol with selenium is supported by complementary mechanisms of action. Selenium is an essential component of several selenoproteins involved in thyroid hormone metabolism and antioxidant defense, including iodothyronine deiodinases and glutathione peroxidases [18]. These enzymes play a critical role in protecting thyroid cells from oxidative stress generated during hormone synthesis. Selenium supplementation has therefore been proposed as an adjunct therapy in autoimmune thyroid disease due to its immunomodulatory and anti-inflammatory properties [19]. On the other hand, myo-inositol functions as a precursor of phosphatidylinositol signaling molecules involved in intracellular signal transduction, including pathways activated by thyroid-stimulating hormone [20]. By enhancing the TSH signaling cascade and improving hydrogen peroxide generation required for thyroid hormone synthesis, myo-inositol may improve thyroid function and sensitivity to TSH. The combined supplementation may therefore act synergistically, improving thyroid function while simultaneously modulating autoimmune processes [20].

Despite the clear reduction in TSH, our analysis did not demonstrate significant differences between treatment groups for fT3 or fT4 levels. This finding is not unexpected, as patients with subclinical hypothyroidism typically maintain normal circulating thyroid hormone concentrations despite elevated TSH levels. The improvement in TSH observed in the MI–Sel group may therefore represent an early physiological response before measurable changes in peripheral hormone levels occur. Additionally, the high heterogeneity observed in the fT4 analysis suggests that methodological differences among the included studies, including patient characteristics, baseline thyroid function, and supplementation regimens, may have influenced the reported results. The subgroup analysis further highlighted this issue, showing that the observational study [18] reported a significant reduction in fT4 levels while the pooled randomized trials did not demonstrate such an effect, indicating that study design may partially explain the observed heterogeneity.

Regarding thyroid autoimmunity, the meta-analysis demonstrated a significant reduction in TgAb levels in the MI–Sel group compared with selenium monotherapy. However, trial sequential analysis indicated that the cumulative evidence did not cross the monitoring boundary for benefit, suggesting that the observed significance may still be susceptible to type I error. Consequently, the potential effect of MI–Sel supplementation on TgAb levels should be interpreted cautiously until additional studies become available. In contrast, no significant effect was observed for TPOAb levels, which are typically considered the most sensitive serological marker of autoimmune thyroiditis. The absence of a clear effect on TPOAb may indicate that the primary benefit of combination therapy is related more to improving thyroid functional regulation rather than strongly suppressing autoimmune activity [20].

Several strengths of this meta-analysis should be acknowledged. First, the study followed a rigorous systematic methodology and included comprehensive searches across major databases. Second, advanced analytical approaches were applied, including trial sequential analysis, leave-one-out sensitivity analyses, Baujat plots, and subgroup analyses, allowing a more consistent assessment of the stability and reliability of the results. Third, heterogeneity was generally low for most outcomes, and sensitivity analyses demonstrated that no single study disproportionately influenced the pooled estimates. These methodological features strengthen the confidence in the observed reduction in TSH levels associated with MI–Sel supplementation.

Nevertheless, several limitations must be considered. The most important limitation is the small number of available studies and the relatively limited sample size. Only three studies met the inclusion criteria, and two of them originated from the same research group [7,17], which may limit the generalizability of the findings. Additionally, one of the included studies had an observational design and was assessed as having a serious risk of bias [18]. Variations in selenium dosage, treatment duration, and baseline patient characteristics may also have contributed to heterogeneity across studies. Furthermore, the small number of included studies limits the ability to reliably assess publication bias, and visual interpretation of funnel plots in such circumstances should be interpreted with caution.

Future research should focus on larger, well-designed randomized controlled trials to confirm the clinical benefits of myo-inositol and selenium combination therapy. These studies should aim to standardize supplementation regimens, evaluate longer follow-up periods, and explore clinically relevant outcomes such as progression from subclinical to overt hypothyroidism, quality of life, and symptom improvement. Additional mechanistic studies may also help clarify how myo-inositol influences thyroid signaling pathways and interacts with selenium-dependent antioxidant systems.

## 5. Conclusions

This meta-analysis found that MI combined with Sel significantly reduces TSH levels compared with Sel alone in patients with Hashimoto’s thyroiditis and subclinical hypothyroidism, suggesting a potential benefit of the combination therapy, although the evidence remains limited. Although a reduction in TgAb levels was observed, TSA indicates that this finding should be interpreted cautiously. Larger, well-designed randomized controlled trials are required to confirm these results and further clarify the effects on thyroid hormones and autoimmune markers.

## Figures and Tables

**Table 1:** Baseline characteristics of the included studies.

Study/Characteristics	Nordio M. & Pajalich 2013 [17]	Nordio M. & Basciani 2017 [7]	Pace et al. 2020 [18]
Study design	RCT	RCT	Observational
Country	Italy	Italy	Italy
No. Patients	48	168	72
No. Males	0	10	5
Age *	37 (MI + Sel) vs. 38 (Sel)	42 (MI + Sel) vs. 42 (Sel)	50 (MI + Sel) vs. 47 (Sel)
Myo-Inositol plus Selenium (Mi + Sel)	24	84	43
Selenium only (Sel)	24	84	29
Dosage	Mi/Sel: 600 mg/83 µg Sel: 83 µg	Mi/Sel: 600 mg/16.6 µg Sel: 83 µg	Mi/Sel: 600 mg/83 µg Sel: 83 µg

* Values are presented as mean age (years).

## Data Availability

Not applicable.

## Supplementary Materials

The following supporting information can be downloaded at: https://www.mdpi.com/article/10.3390/jcm15093179/s1, PRISMA 2020 checklist. Figure S1. LOO sensitivity analysis for TSH levels. Figure S2. Baujat plot of TSH levels. Figure S3. LOO sensitivity analysis for the comparison of MI-SEL and SEL in terms of ft4 levels. Figure S4. Baujat plot identifying sources of heterogeneity in terms of ft4 levels. Figure S5. LOO sensitivity analysis for the effect of MI-SEL vs. SEL in ft3 levels. Figure S6. Baujat plot identifying sources of heterogeneity in fT3 levels. Figure S7. LOO sensitivity analysis for the comparison of MI-SEL and SEL on TgAb levels. Figure S8. Baujat plot identifying sources of heterogeneity in TgAb levels. Figure S9. LOO sensitivity analysis for the effect of MI-SEL vs. SEL on TPOAb levels. Figure S10. Baujat plot identifying sources of heterogeneity. Figure S11. Subgroup analysis of TSH reduction by study design. Figure S12. Subgroup analysis of fT4 levels by study design. Figure S13. Subgroup analysis of fT3 levels by study design. Figure S14. ROB assessment (ROBINS-I) summary for the observational study. Figure S15. ROB assessment (RoB2.0) summary for the RCT studies. Figure S16. Contour-enhanced trim-and-fill funnel plot for TSH. Figure S17. Contour-enhanced trim-and-fill funnel plot for fT4. Figure S18. Contour-enhanced trim-and-fill funnel plot for fT3. Figure S19. Contour-enhanced trim-and-fill funnel plot for TGAB. Figure S20. Contour-enhanced trim-and-fill funnel plot for TPOAB.
