# Supplementary material for: Myo-Inositol Plus Selenium vs. Selenium Alone in Hashimoto’s Thyroiditis with Subclinical Hypothyroidism: A Systematic Review and Updated Meta-Analysis with Trial Sequential Analysis
Source: J Clin Med. 2026 Apr 22;15(9):3179. doi: 10.3390/jcm15093179 (PMC13164135; doi:10.3390/jcm15093179)
Supplement: Supplementary file 1 [file jcm-15-03179-s001.zip › jcm-4249048-supplementary.pdf]

PRISMA 2020 Checklist

Section and Topic	Item #	Checklist item	Location where item is reported
**TITLE**			
Title	1	Identify the report as a systematic review.	Title, 1-2
**ABSTRACT**			
Abstract	2	See the PRISMA 2020 for Abstracts checklist.	Abstract, 12-34
**INTRODUCTION**			
Rationale	3	Describe the rationale for the review in the context of existing knowledge.	Introduction, 38-70
Objectives	4	Provide an explicit statement of the objective(s) or question(s) the review addresses.	Introduction, 71-76
**METHODS**			
Eligibility criteria	5	Specify the inclusion and exclusion criteria for the review and how studies were grouped for the syntheses.	Methods, 77-101
Information sources	6	Specify all databases, registers, websites, organisations, reference lists and other sources searched or consulted to identify studies. Specify the date when each source was last searched or consulted.	Methods, 102-113
Search strategy	7	Present the full search strategies for all databases, registers and websites, including any filters and limits used.	Methods, 102-113
Selection process	8	Specify the methods used to decide whether a study met the inclusion criteria of the review, including how many reviewers screened each record and each report retrieved, whether they worked independently, and if applicable, details of automation tools used in the process.	Methods, 102-113
Data collection process	9	Specify the methods used to collect data from reports, including how many reviewers collected data from each report, whether they worked independently, any processes for obtaining or confirming data from study investigators, and if applicable, details of automation tools used in the process.	Methods, 102-113
Data items	10a	List and define all outcomes for which data were sought. Specify whether all results that were compatible with each outcome domain in each study were sought (e.g., for all measures, time points, analyses), and if not, the methods used to decide which results to collect.	Methods, 114-116
	10b	List and define all other variables for which data were sought (e.g., participant and intervention characteristics, funding sources). Describe any assumptions made about any missing or unclear information.	Methods, 114-116
Study risk of bias assessment	11	Specify the methods used to assess risk of bias in the included studies, including details of the tool(s) used, how many reviewers assessed each study and whether they worked independently, and if applicable, details of automation tools used in the process.	Methods, 117-130
Effect measures	12	Specify for each outcome the effect measure(s) (e.g., risk ratio, mean difference) used in the synthesis or presentation of results.	Methods, 131-144
Synthesis methods	13a	Describe the processes used to decide which studies were eligible for each synthesis (e.g., tabulating the study intervention characteristics and comparing against the planned groups for each synthesis (item #5)).	Methods, 131-144
	13b	Describe any methods required to prepare the data for presentation or synthesis, such as handling of missing summary statistics, or data conversions.	Methods, 131-144
	13c	Describe any methods used to tabulate or visually display results of individual studies and syntheses.	Methods, 131-144
	13d	Describe any methods used to synthesize results and provide a rationale for the choice(s). If meta-analysis was performed, describe the model(s), method(s) to identify the presence and extent of statistical heterogeneity, and software package(s) used.	Methods, 131-144

PRISMA 2020 Checklist

Section and Topic	Item #	Checklist item	Location where item is reported
	13e	Describe any methods used to explore possible causes of heterogeneity among study results (e.g., subgroup analysis, meta-regression).	Methods, 131-144
	13f	Describe any sensitivity analyses conducted to assess robustness of the synthesized results.	Methods, 131-144
Reporting bias assessment	14	Describe any methods used to assess risk of bias due to missing results in a synthesis (arising from reporting biases).	Methods, 117-130
Certainty assessment	15	Describe any methods used to assess certainty (or confidence) in the body of evidence for an outcome.	Methods, 117-130
**RESULTS**			
Study selection	16a	Describe the results of the search and selection process, from the number of records identified in the search to the number of studies included in the review, ideally using a flow diagram.	Results, 154-161
	16b	Cite studies that might appear to meet the inclusion criteria, but which were excluded, and explain why they were excluded.	Results, 154-161
Study characteristics	17	Cite each included study and present its characteristics.	Results, 164-165
Risk of bias in studies	18	Present assessments of risk of bias for each included study.	Results, 323-331
Results of individual studies	19	For all outcomes, present, for each study: (a) summary statistics for each group (where appropriate) and (b) an effect estimate and its precision (e.g., confidence/credible interval), ideally using structured tables or plots.	Results, 166-322
Results of syntheses	20a	For each synthesis, briefly summarise the characteristics and risk of bias among contributing studies.	Results, 166-322
	20b	Present results of all statistical syntheses conducted. If meta-analysis was done, present for each the summary estimate and its precision (e.g., confidence/credible interval) and measures of statistical heterogeneity. If comparing groups, describe the direction of the effect.	Results, 166-322
	20c	Present results of all investigations of possible causes of heterogeneity among study results.	Results, 166-322
	20d	Present results of all sensitivity analyses conducted to assess the robustness of the synthesized results.	Results, 166-322
Reporting biases	21	Present assessments of risk of bias due to missing results (arising from reporting biases) for each synthesis assessed.	Results, 323-331
Certainty of evidence	22	Present assessments of certainty (or confidence) in the body of evidence for each outcome assessed.	Results, 323-331
**DISCUSSION**			
Discussion	23a	Provide a general interpretation of the results in the context of other evidence.	Discussion, 351-420
	23b	Discuss any limitations of the evidence included in the review.	Discussion, 421-430
	23c	Discuss any limitations of the review processes used.	Discussion, 421-430

Section and Topic	Item #	Checklist item	Location where item is reported
	23d	Discuss implications of the results for practice, policy, and future research.	Discussion, 431-437
**OTHER INFORMATION**			
Registration and protocol	24a	Provide registration information for the review, including register name and registration number, or state that the review was not registered.	Methods, 77-101
	24b	Indicate where the review protocol can be accessed, or state that a protocol was not prepared.	Methods, 77-101
	24c	Describe and explain any amendments to information provided at registration or in the protocol.	Methods, 77-101
Support	25	Describe sources of financial or non-financial support for the review, and the role of the funders or sponsors in the review.	Under Conclusion, 446-447
Competing interests	26	Declare any competing interests of review authors.	Under Conclusion, 448
Availability of data, code and other materials	27	Report which of the following are publicly available and where they can be found: template data collection forms; data extracted from included studies; data used for all analyses; analytic code; any other materials used in the review.	Under Conclusion, 456

From: Page MJ, McKenzie JE, Bossuyt PM, Boutron I, Hoffmann TC, Mulrow CD, et al. The PRISMA 2020 statement: an updated guideline for reporting systematic reviews. BMJ 2021;372:n71. doi: 10.1136/bmj.n71. This work is licensed under CC BY 4.0. To view a copy of this license, visit https://creativecommons.org/licenses/by/4.0/

Supplementary Results

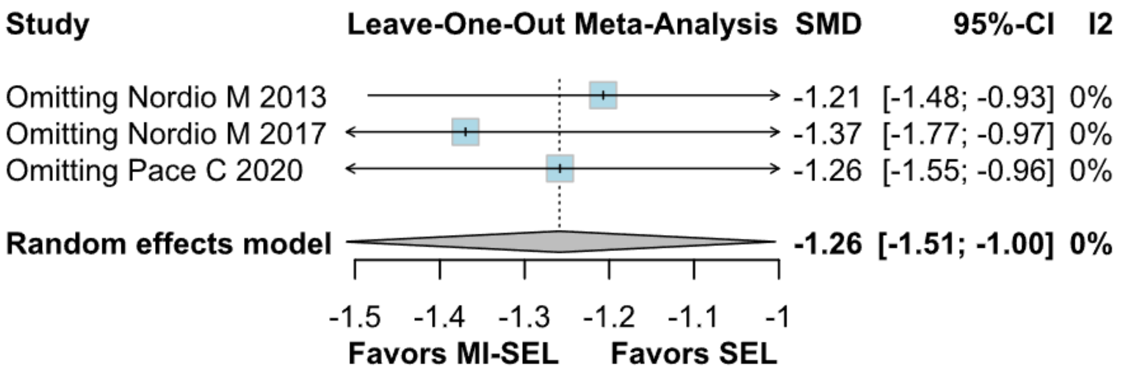

Supplementary Figure S1. LOO sensitivity analysis for TSH levels. In terms of TSH levels, the overall effect size remained consistent across all iterations and the result remained significant in all cases. Heterogeneity remained zero throughout all iterations.

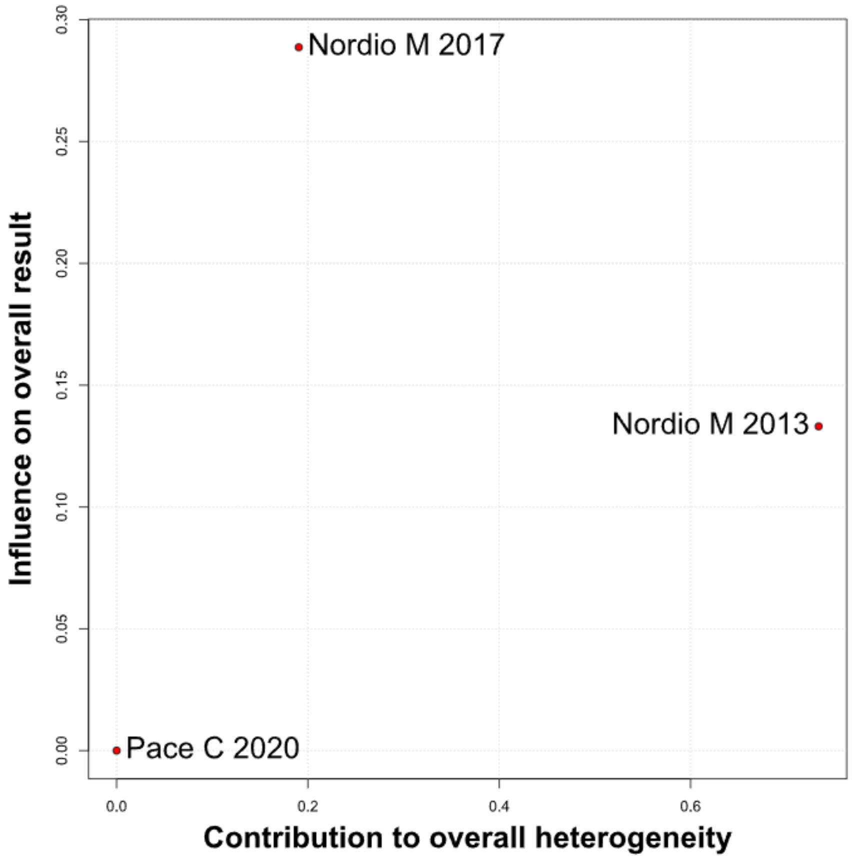

Supplementary Figure S2. Baujat plot of TSH levels. The studies by Nordio M & Basciani 2017 and Nordio M & Pajalich 2013 were discovered as potentially influential, contributing substantially to the overall result and heterogeneity.

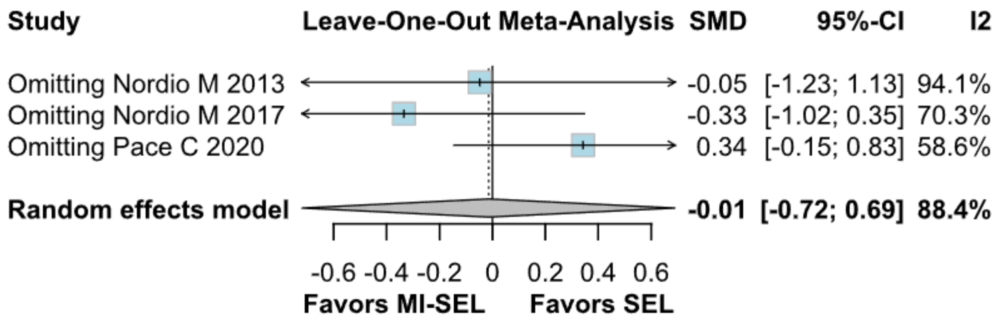

Supplementary Figure S3. LOO sensitivity analysis for the comparison of MI-SEL and SEL in terms of ft4 levels. The results demonstrate that the overall non-significant effect remains stable regardless of which individual study is omitted.

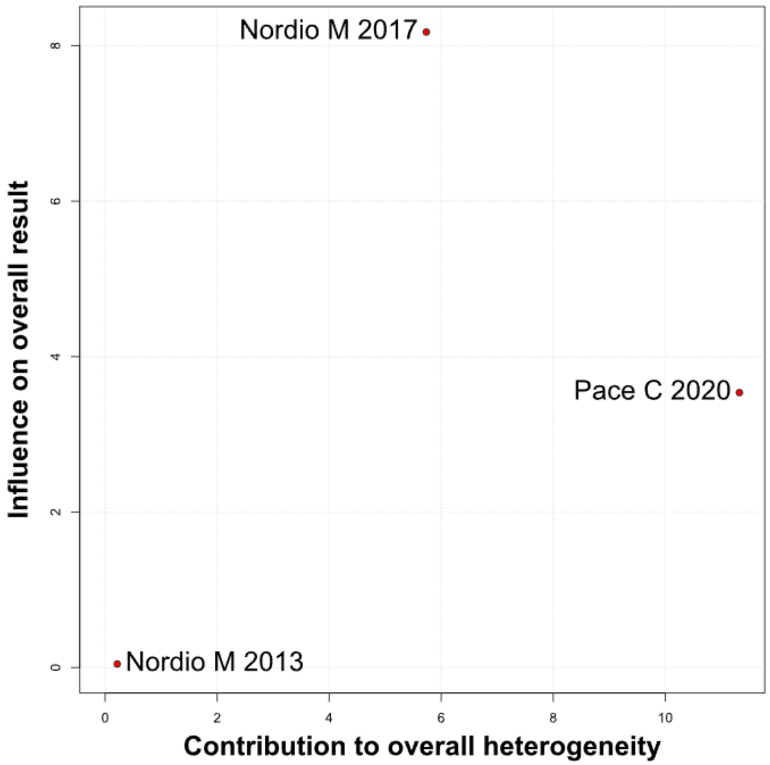

Supplementary Figure S4. Baujat plot identifying sources of heterogeneity in terms of ft4 levels. Nordio M & Basciani 2017 showed the highest

influence on the overall pooled result, while Pace et al. 2020 contribute most significantly to the overall statistical heterogeneity.

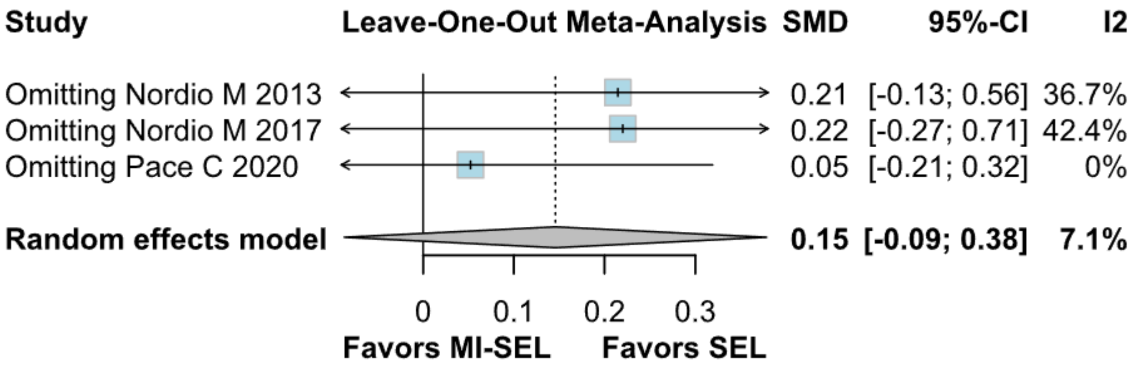

Supplementary Figure S5. LOO sensitivity analysis for the effect of MI-SEL vs. SEL in ft3 levels. The stability of the results is confirmed, as the omission of any single study does not shift the pooled estimate to statistical significance. Notably, omitting Pace et al. 2020 reduces heterogeneity (I2) to 0%, identifying it as the primary source of variance.

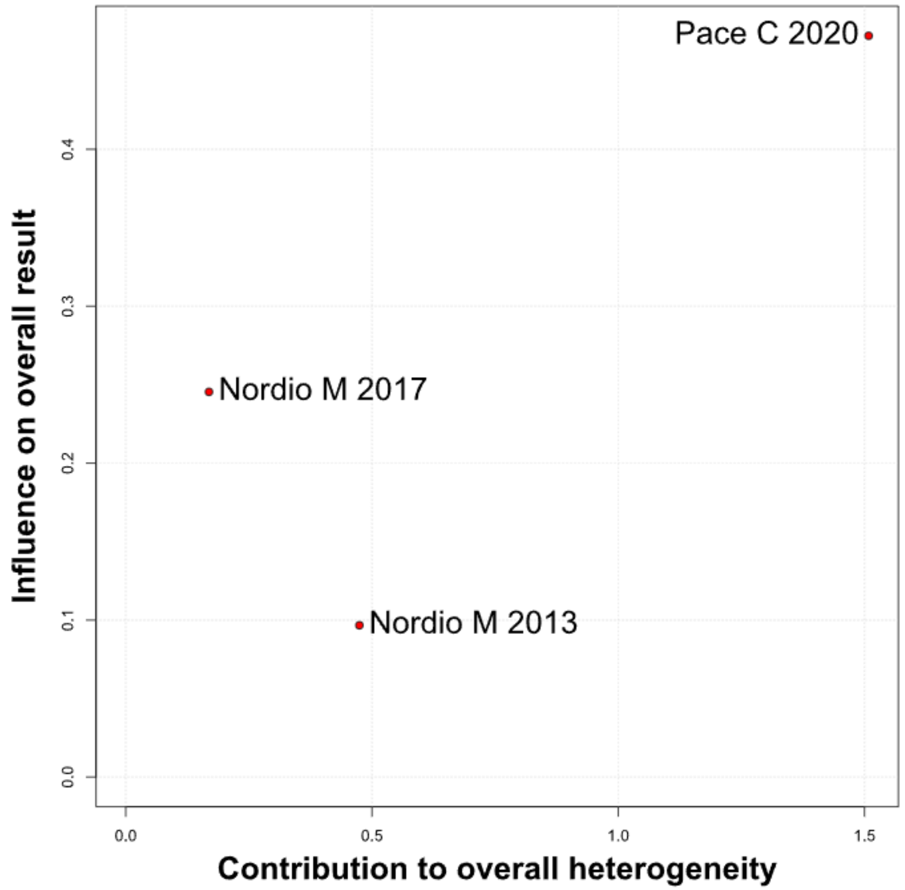

Supplementary Figure S6. Baujat plot identifying sources of heterogeneity in fT3 levels. Nordio M & Basciani 2017 show the highest influence on the overall pooled result, while Pace et al. 2020 contribute most significantly to the overall statistical heterogeneity.

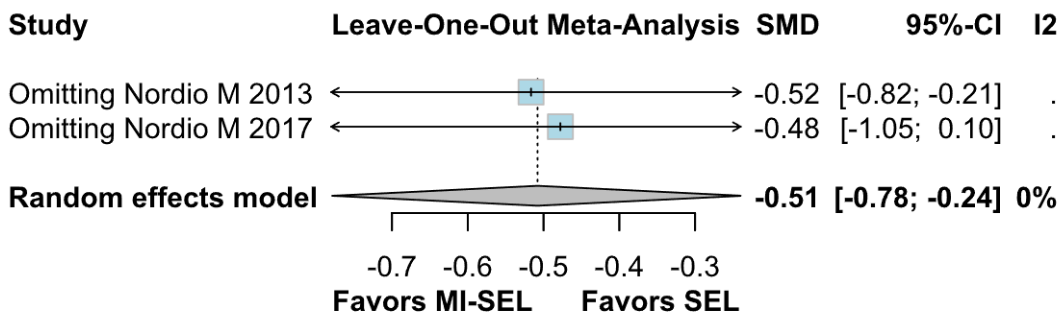

Supplementary Figure S7. LOO sensitivity analysis for the comparison of MI-SEL and SEL on TgAb levels. The results demonstrate that the overall significant effect remains stable regardless of which individual study is omitted.

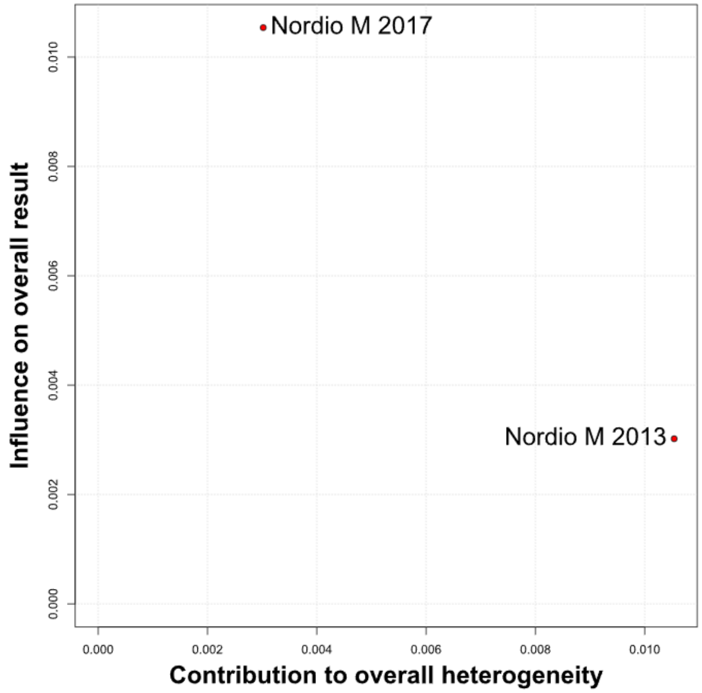

Supplementary Figure S8. Baujat plot identifying sources of heterogeneity in TgAb levels. Nordio M & Basciani 2017 show the highest influence on the overall pooled result, while Nordio M & Pajalich 2013 contribute most significantly to the overall statistical heterogeneity.

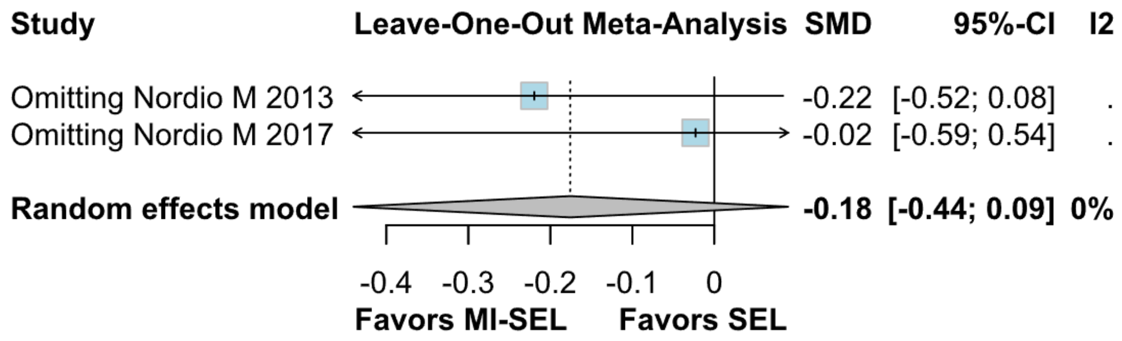

Supplementary Figure S9. LOO sensitivity analysis for the effect of MI-SEL vs. SEL on TPOAb levels. The stability of the results is confirmed, as the omission of any single study does not shift the pooled estimate to statistical significance.

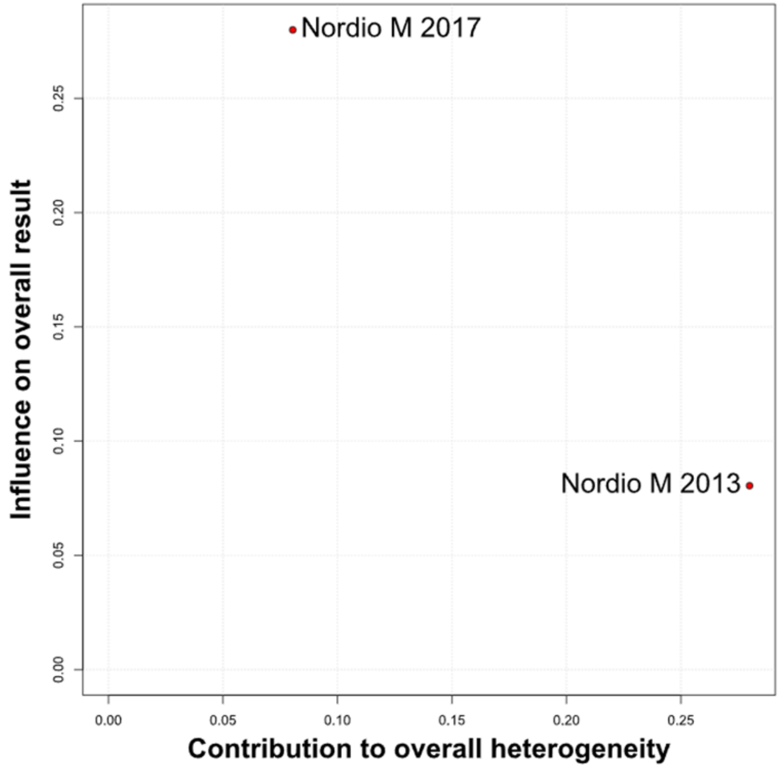

Supplementary Figure S10. Baujat plot identifying sources of heterogeneity. Nordio M & Basciani 2017 show the highest influence on the overall pooled result, while Nordio M & Pajalich 2013 contribute most significantly to the overall statistical heterogeneity.

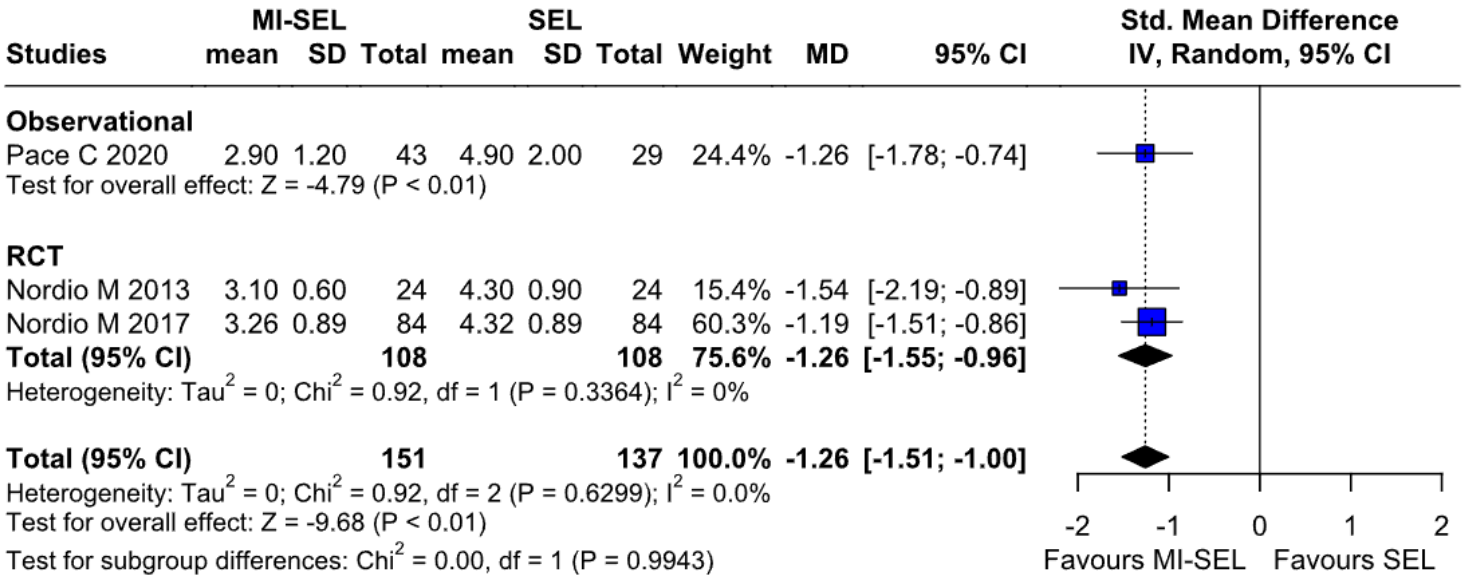

Supplementary Figure S11. Subgroup analysis of TSH reduction by study design. The combined MI-SEL treatment demonstrated a significant and identical effect size across both subgroups (SMD: -1.26; 95% CI[-1.51;-1.00]; p<0.01), with no significant difference between study methodologies (subgroup p=0.9943).

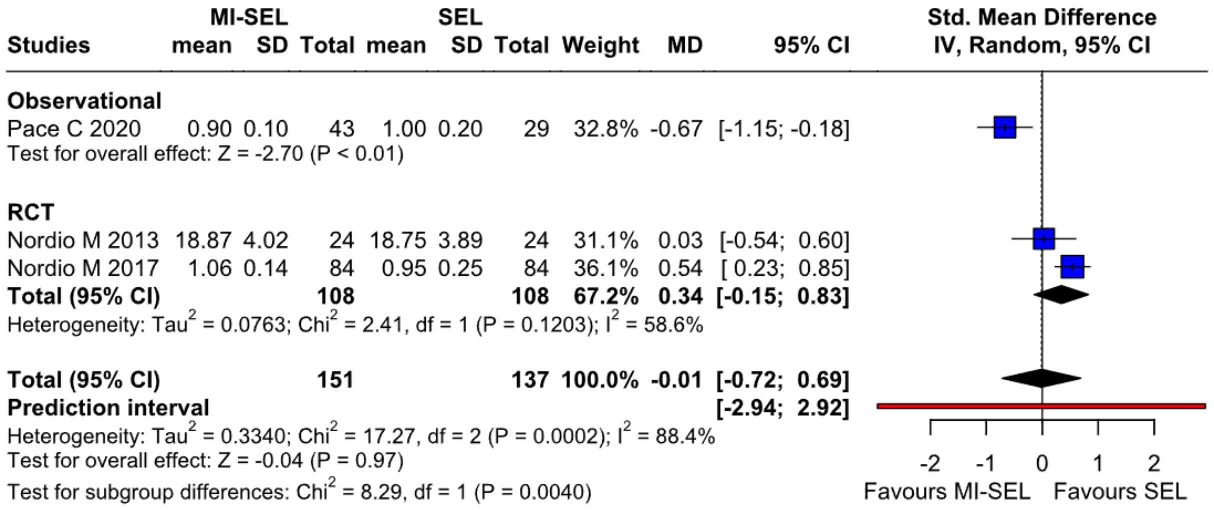

Supplementary Figure S12. Subgroup analysis of fT4 levels by study design. While the combined treatment (MI-SEL) showed a significant effect in the observational study, no significant difference was observed in the RCT subgroup. The overall pooled effect was non-significant (SMD: -0.01; 95% CI[-0.72; 0.69]; p=0.97), with significant subgroup differences identified (p=0.004).

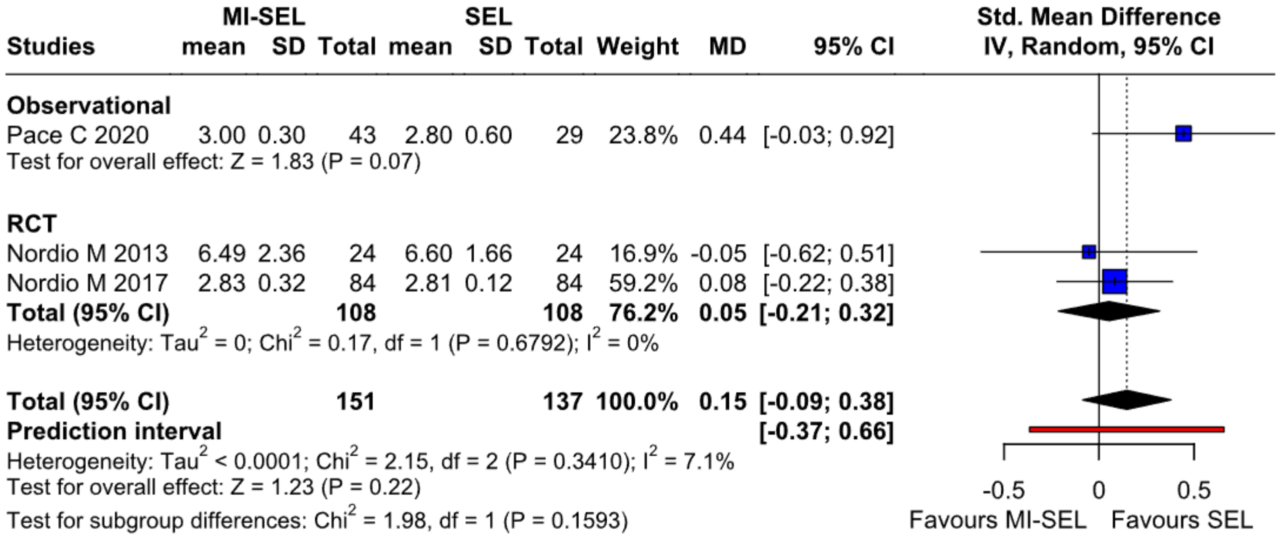

Supplementary Figure S13. Subgroup analysis of fT3 levels by study design. No significant difference was observed between MI-SEL and SEL groups (Overall SMD 0.15; 95% CI[-0.09; 0.38]; p=0.22). Results remained consistent across both RCT and observational subgroups (p=0.16 for subgroup differences), with negligible overall heterogeneity (I2=7.1%).

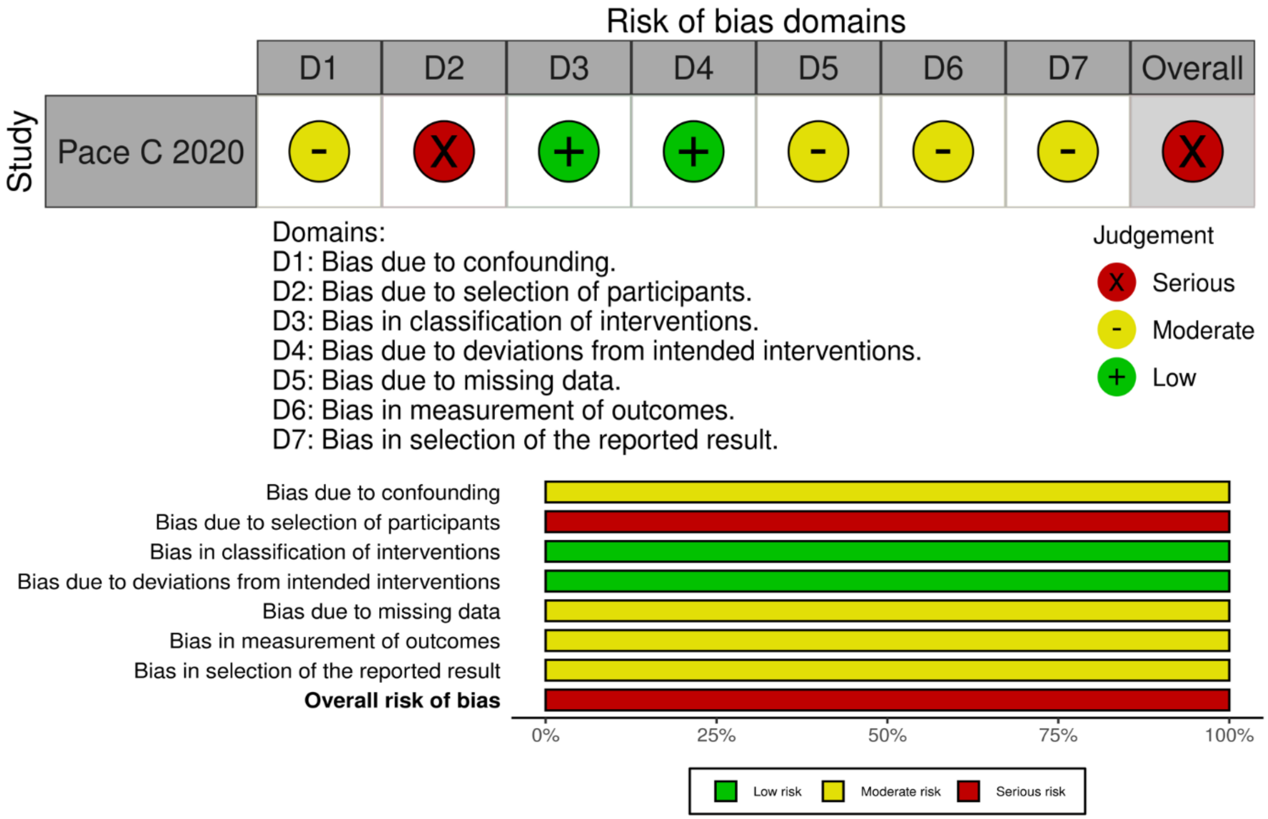

Supplementary Figure S14. ROB assessment (ROBINS-I) summary for the observational study.

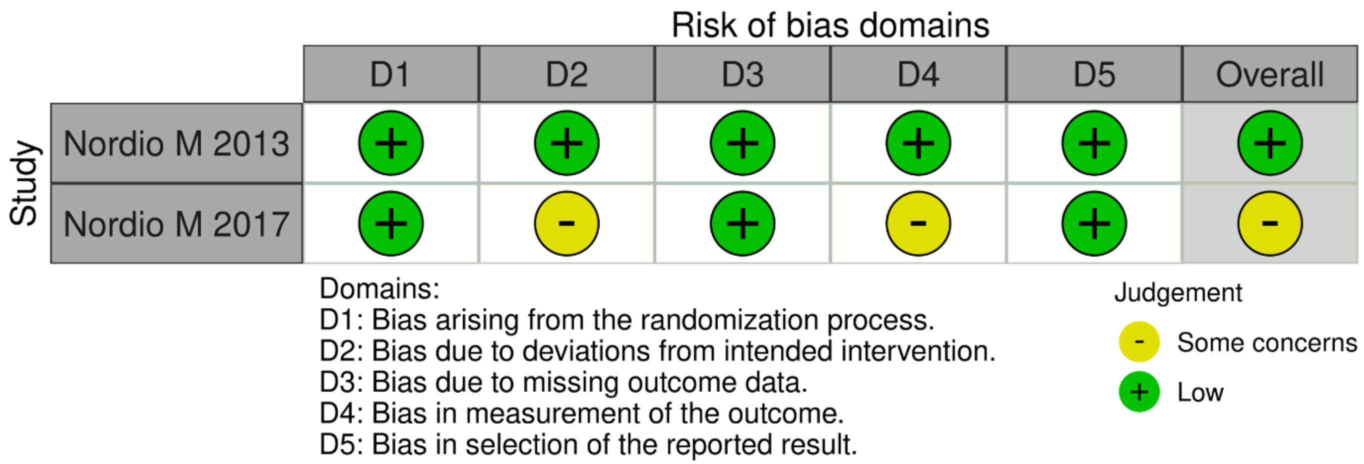

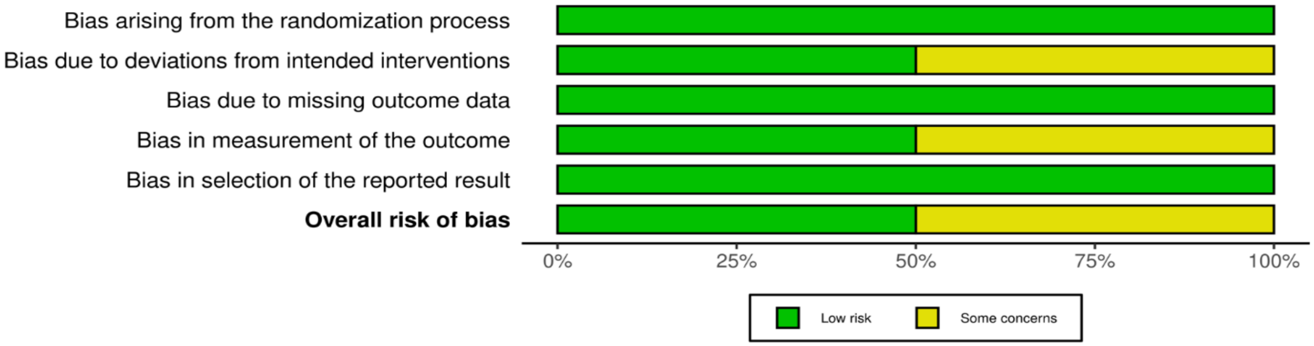

Supplementary Figure S15. ROB assessment (RoB2.0) summary for the RCT studies.

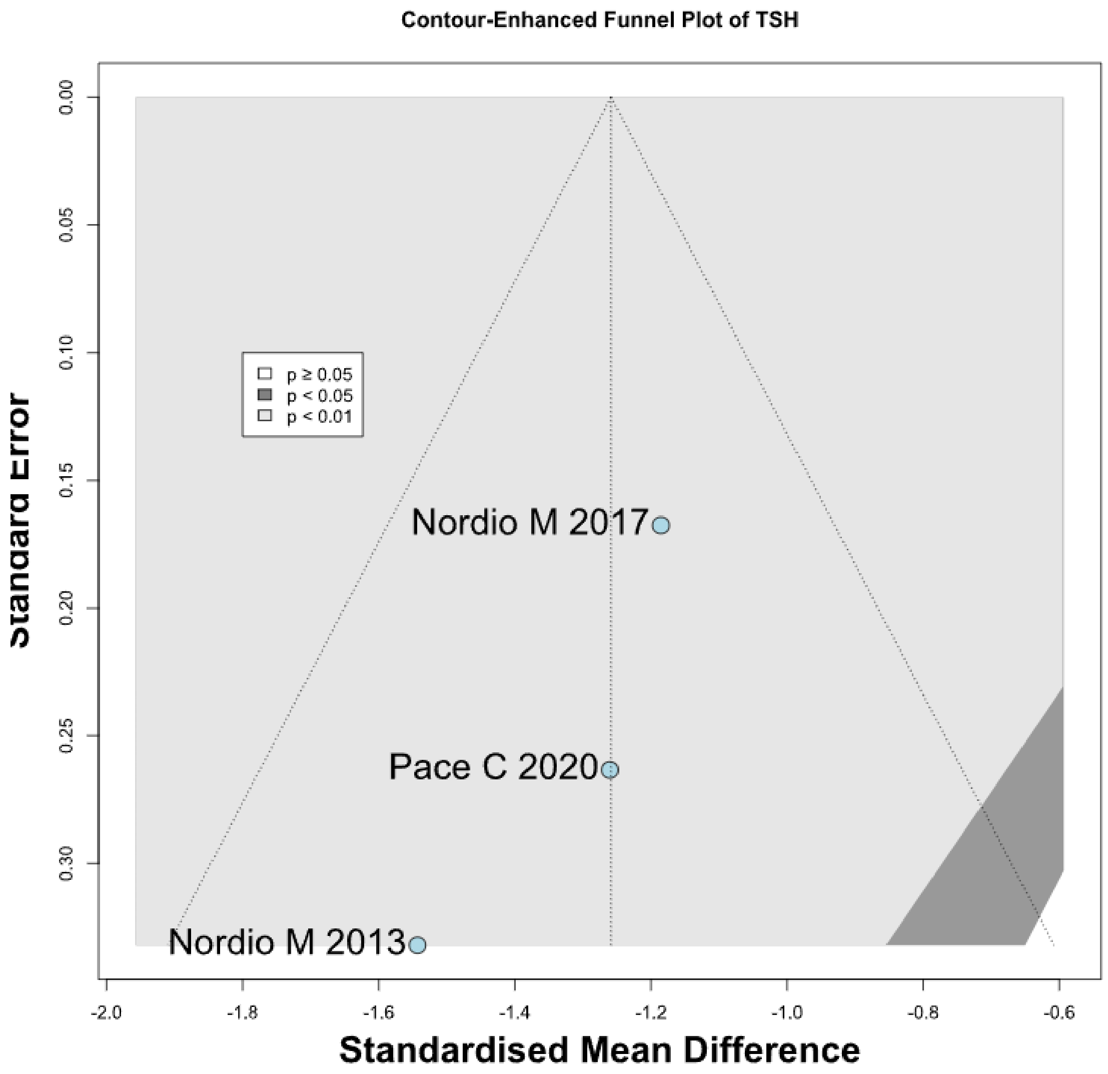

Supplementary Figure S16. Contour-enhanced trim-and-fill funnel plot for TSH. The plot illustrates individual study weights against point estimates.

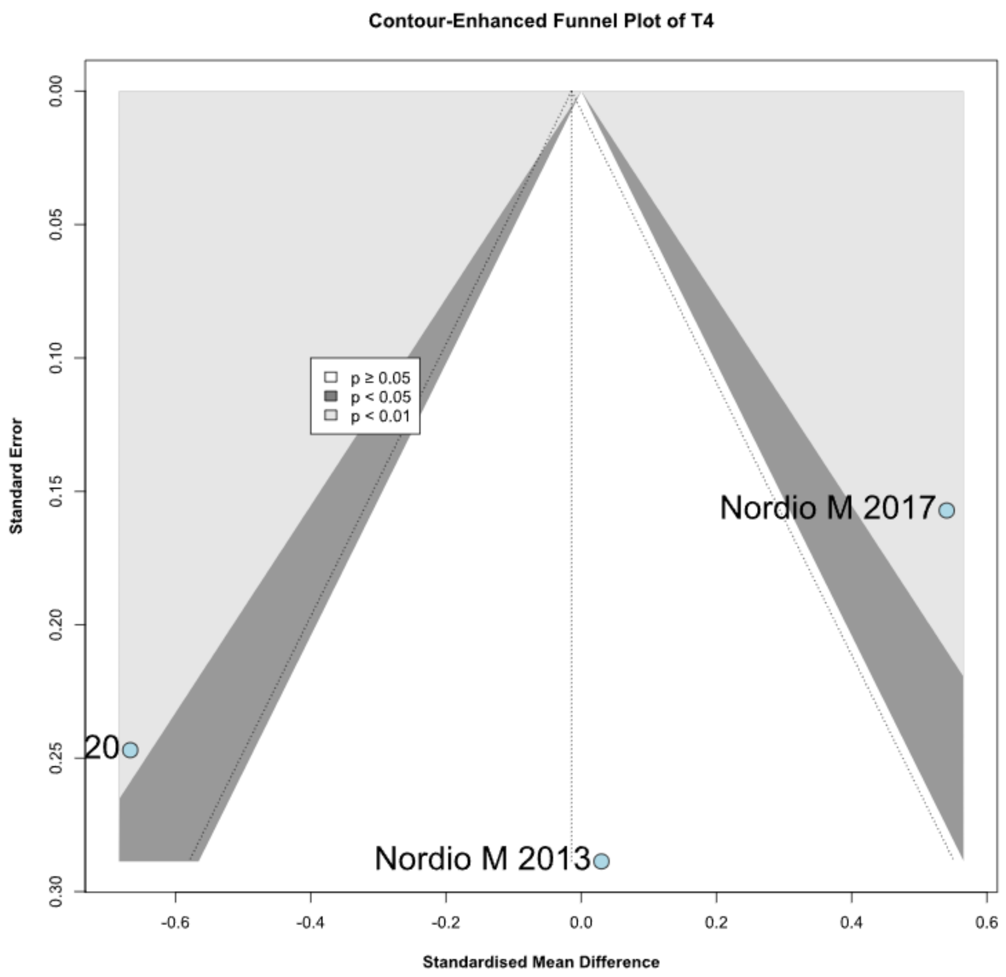

Supplementary Figure S17. Contour-enhanced trim-and-fill funnel plot for fT4. The plot illustrates individual study weights against point estimates.

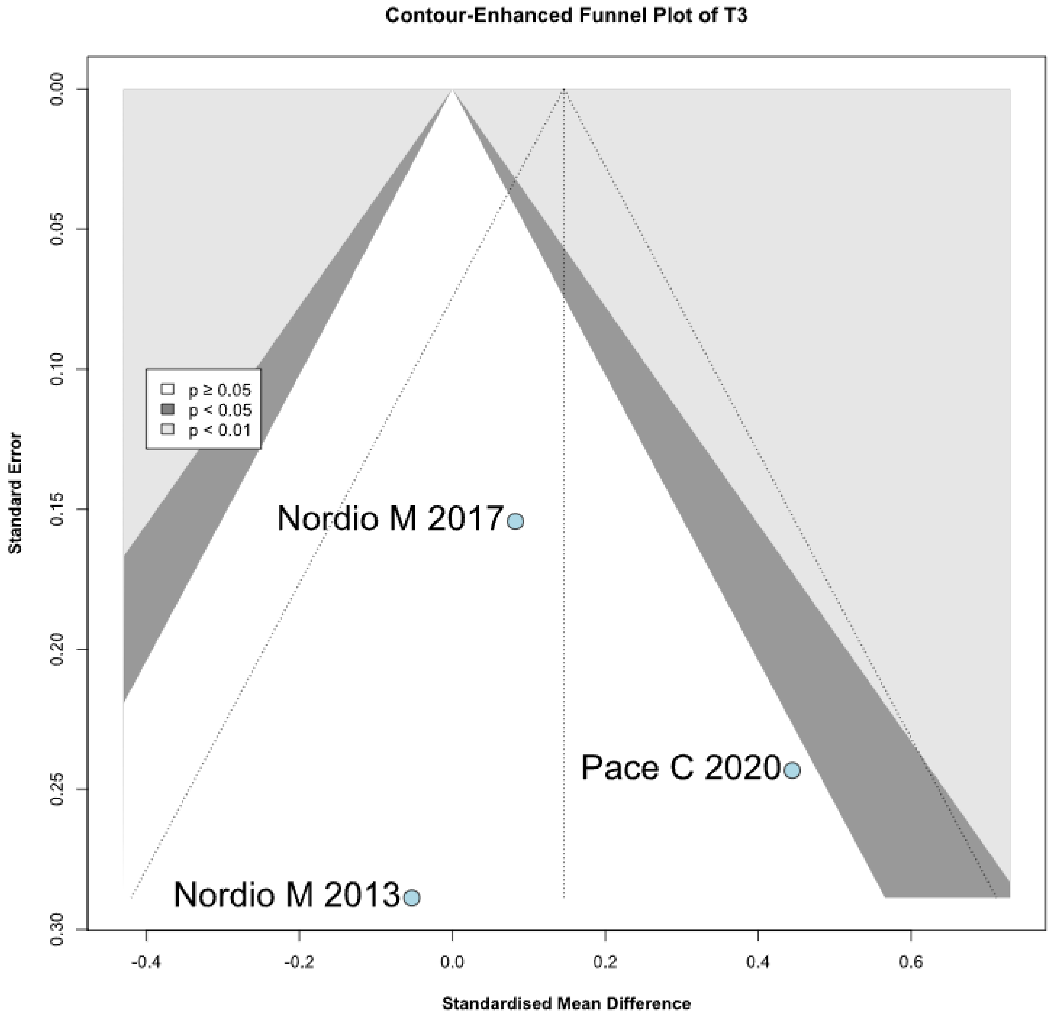

Supplementary Figure S18. Contour-enhanced trim-and-fill funnel plot for fT3. The plot illustrates individual study weights against point estimates.

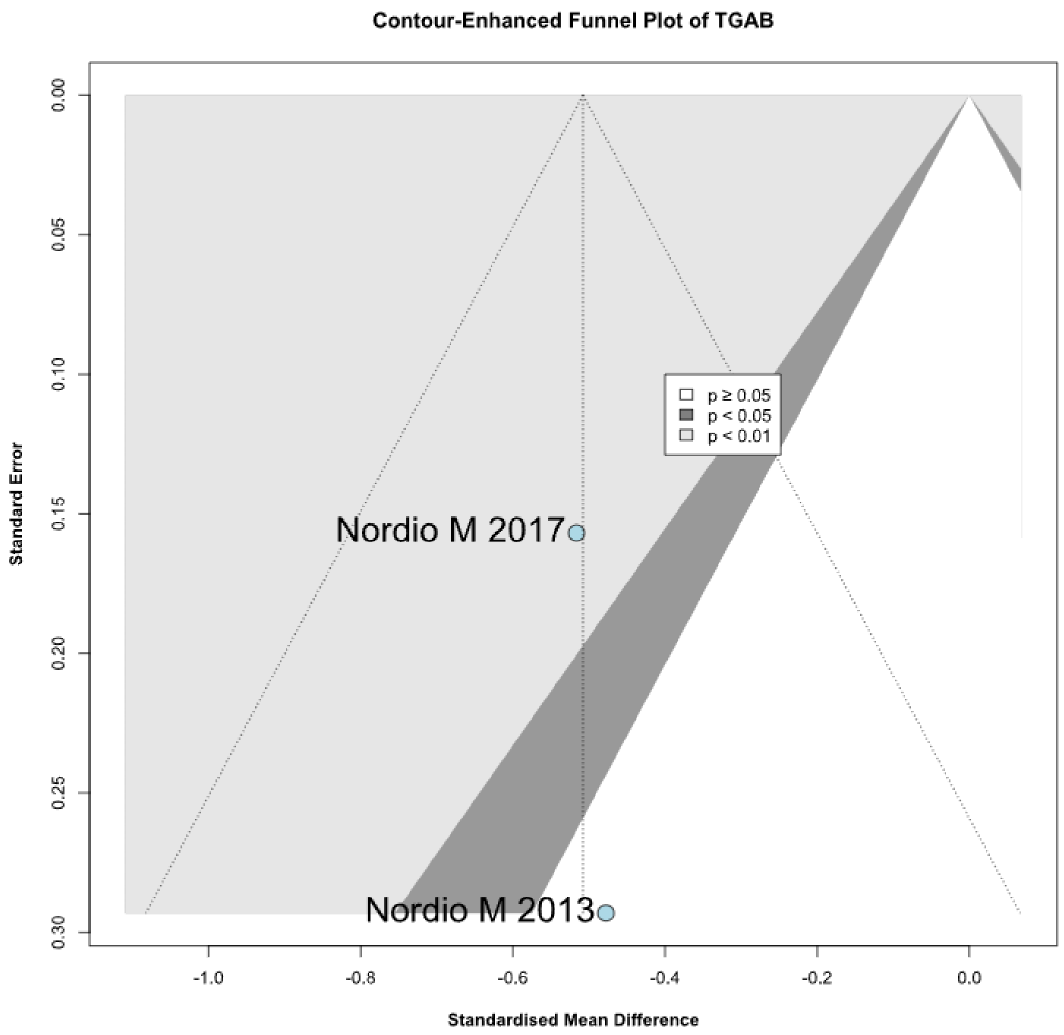

Supplementary Figure S19. Contour-enhanced trim-and-fill funnel plot for TGAB. The plot illustrates individual study weights against point estimates.

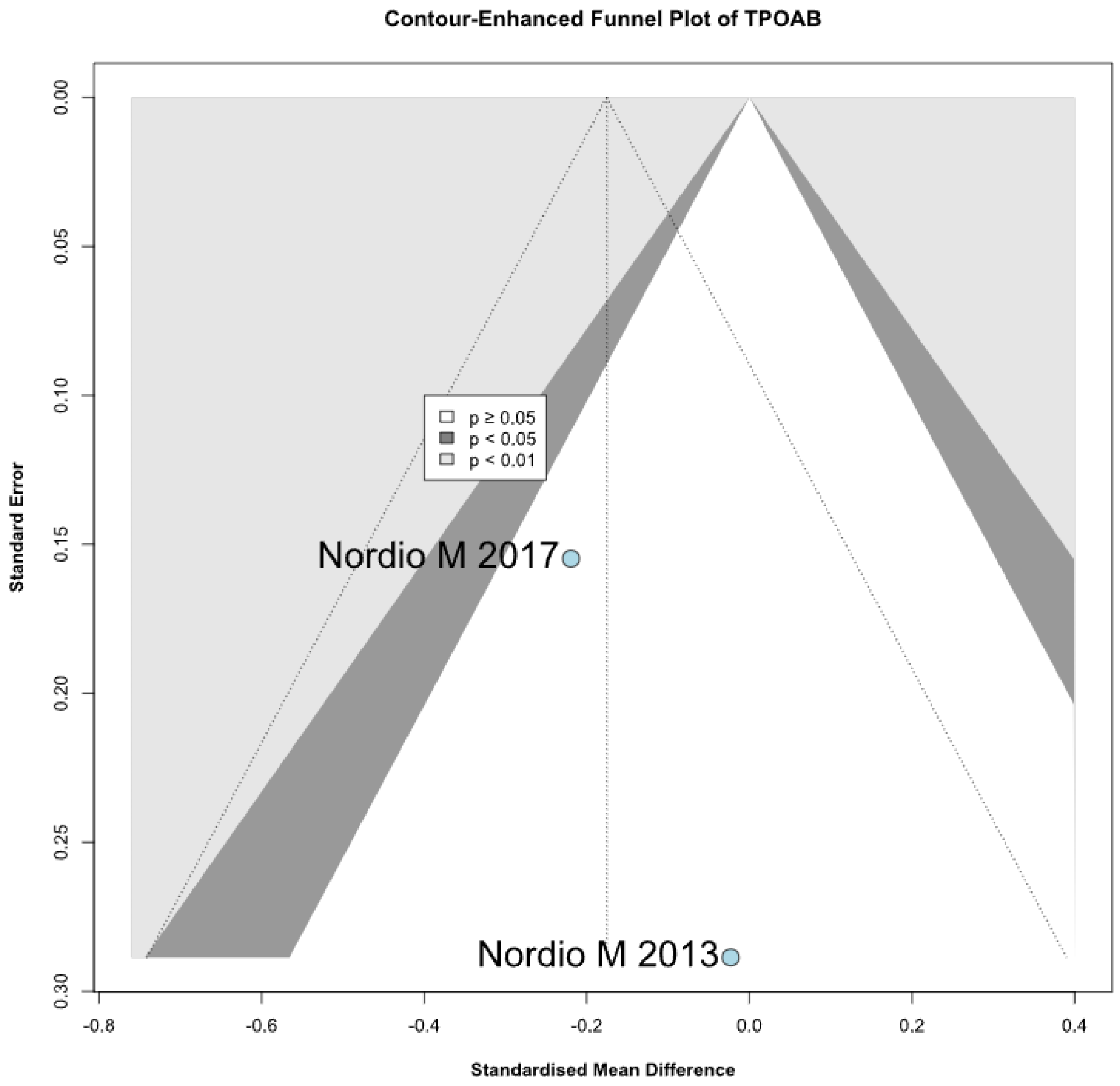

Supplementary Figure S20. Contour-enhanced trim-and-fill funnel plot for TPOAB. The plot illustrates individual study weights against point estimates.
